# Label-free *in vitro* toxicity and uptake assessment of citrate stabilised gold nanoparticles in three cell lines

**DOI:** 10.1186/1743-8977-10-50

**Published:** 2013-10-09

**Authors:** Melissa A Vetten, Nonhlanhla Tlotleng, Delia Tanner Rascher, Amanda Skepu, Frankline K Keter, Kailen Boodhia, Leigh-Anne Koekemoer, Charlene Andraos, Robert Tshikhudo, Mary Gulumian

**Affiliations:** 1 Toxicology Section, National Institute for Occupational Health (NIOH), P O Box 4788, Johannesburg 2000, South Africa; 2 Haematology and Molecular Medicine, School of Pathology, University of the Witwatersrand, Johannesburg 2000, South Africa; 3 Nanotechnology Innovation Centre (NIC), Advanced Materials Division, Mintek, Private Bag X 3015, Randburg 2125, South Africa

**Keywords:** Gold nanoparticles, Cell impedance, *in vitro* toxicity, Hyperspectral imaging, Uptake, Label-free

## Abstract

**Background:**

Reliable *in vitro* toxicity testing is needed prior to the commencement of *in vivo* testing necessary for hazard identification and risk assessment of nanoparticles. In this study, the cytotoxicity and uptake of 14 nm and 20 nm citrate stabilised gold nanoparticles (AuNPs) in the bronchial epithelial cell line BEAS-2B, the Chinese hamster ovary cell line CHO, and the human embryonic kidney cell line HEK 293 were investigated.

**Methods:**

Cytotoxicity of the AuNPs was assessed via traditional XTT-, LDH-, and ATP-based assays, followed by cell impedance studies. Dark-field imaging and hyperspectral imaging were used to confirm the uptake of AuNPs into the cells.

**Results:**

Interference of the AuNPs with the XTT- and ATP-based assays was overcome through the use of cell impedance technology. AuNPs were shown to be relatively non-toxic using this methodology; nevertheless CHO cells were the most sensitive cell type with 20 nm AuNPs having the highest toxicity. Uptake of both 14 nm and 20 nm AuNPs was observed in all cell lines in a time- and cell type-dependent manner.

**Conclusions:**

Using the cell impedance and dark-field hyperspectral imaging technologies, it was possible to study the toxicity of AuNPs in different cell lines and show that these cells could internalize AuNPs with their subsequent intracellular aggregation. It was also possible to show that this toxicity would not correlate with the level of uptake but it would correlate with cell-type and the size of the AuNPs. Therefore, these two label-free methodologies used in this study are suitable for *in vitro* studies on the effects of AuNPs, and could present themselves as appropriate and valuable methodologies for future nanoparticle toxicity and uptake studies.

## Background

As the field of nanotechnology develops, studies to investigate the toxicity of engineered nanoparticles become critically important. A tiered approach for nanoparticle toxicity tests has been proposed [1] whereby in-depth physicochemical characterisation of engineered nanomaterials is performed followed by a tier of *in vitro* testing. Positive, consistent results from *in vitro* studies lead to a higher tier of *in vivo* testing, and eventually to hazard identification and classification. Therefore, it is imperative that the *in vitro* toxicity assessment provides reliable data before the commencement of time-consuming and expensive *in vivo* studies.

The traditional cytotoxicity assays that are frequently used to assess *in vitro* toxicity of AuNPs include the 3-(4,5-dimethylthiazol-2-yl)-2,5-diphenyltetrazolium bromide (MTT) assay which is based on the reduction of the tetrazolium salt by the mitochondria to form a colorimetric product, the release of lactate dehydrogenase (LDH), a marker of membrane integrity, and also intracellular adenosine triphosphate (ATP) levels, a marker of metabolically active cells. A previous study which investigated the size-dependent cytotoxicity of 0.8 nm, 1.2 nm, 1.4 nm, 1.8 nm, and 15 nm AuNPs in the cell lines L929, HeLa, J774A1, and SK-Mel-28 found that nanoparticles in the 0.8 – 1.8 nm range were highly toxic whilst the 15 nm nanoparticle was shown to be relatively nontoxic with the MTT colorimetric assay [2]. AuNPs of 20 nm and 100 nm did not affect the viability of human retina microvascular endothelial cells as determined by the MTT assay *in vitro*[3], in addition no toxicity of the 20 nm AuNPs in the rat astrocyte cell line CTX-TNA2 or in human retinoblastoma cells SNUOT-Rb1 was shown. Further cytotoxicity studies with the MTT assay have shown no effect on the viability of the cells exposed to citrate stabilized AuNPs of sizes 3 nm, 5 nm, 12 nm, 17 nm, 37 nm, 50 nm, and 100 nm in HeLa cells [4], no effect of 20 nm AuNPs on the viability of human dermal fibroblasts-fetal cells [5] and no effect of 9.5 nm, 11.19 nm, and 25 nm citrate stabilised AuNPs on A549 and NCIH441 cells with the exception of the 11.19 nm and 25 nm at the highest concentration in A549 cells which displayed mild toxicity [6]. Yet, in this latter study, a dose- and time-dependant increase of LDH release was observed [6]. Using 3-(4,5-dimethylthiazole-2-yl)-5-(3-carboxymethoxyphenyl)-2-(4-sulfophenyl)-2H-tetrazoliumin (MTS), a tetrazolium salt similar to MTT, no toxicity was observed in human cerebral microvascular endothelial (hcMEC/D3) cells and human dermal microvascular endothelial (HDMEC) cells incubated with 10 nm, 11 nm, and 25 nm AuNPs, however, once again, a dose-dependent release of LDH was seen following incubation with the nanoparticles [7]. A more recent study demonstrated that 21 nm citrate-stabilised AuNPs were toxic to PC-3 and MCF-7 cells in a time- and dose dependent manner based on both MTT and LDH release [8]. Finally, the 17 nm citrate capped AuNPs had shown toxicity to A549 cells assessed by both MTT and LDH assays as well as by the ATP depletion measurements [9].

Despite differences in the size of the AuNPs used as well as the cell types implemented, results presented in the literature may not allow one to draw meaningful conclusions as to the toxicity or lack thereof for citrate-capped AuNPs when assessed by the MTT, LDH, and ATP tests. An important aspect about these toxicity test systems is that they rely on absorbance, fluorescence or luminescence changes of the final product. As AuNPs absorb light in the visible region (~520 nm), their interference with these colorimetric, flourometric, and luminometric test systems have been shown to be of great concern [10-12]. In addition to toxicity, it is also necessary to investigate the uptake of nanoparticles (NPs) as their intracellular fate could influence cell toxicity and cellular functioning. Using Transmission Electron Microscopy (TEM), it was shown that the incubation of AuNPs of diameters of 14, 30, 50, 74, and 100 nm with the mammalian cell line HeLa could produce the cellular uptake of all sizes of AuNPs, but the maximum uptake was attained with the 50 nm AuNPs [13]. With confocal microscopy, an increased accumulation within U937 cells was shown for both 20 nm and 30 nm AuNPs as compared to the 45 nm AuNP counterparts [14]. Implementing both Scanning Electron Microscopy (SEM) and TEM, cellular uptake and intracellular distribution of 13 and 45 nm AuNPs in CF-31 cells could be observed and NPs were found to be located in intracellular vacuoles. The number of particles per vacuole was however greater for the 13 nm AuNPs, even though the vacuole number per cell for both sizes of AuNPs was similar [15]. Lastly, TEM was used to show the uptake of 15 nm AuNPs by triple cell co-culture which consisted of human blood monocyte derived macrophages and dendritic cells as well as the A549 human alveolar epithelial cells [16].

In addition, AuNP toxicity in relation to cellular uptake has been studied. For example, it was found that 18 nm citrate-capped AuNPs were not toxic at ~ 100 μM to K562 cells assayed by MTT despite their increased cellular uptake which was assessed by TEM [17]. On the other hand, 10 nm and 25 nm citrate capped AuNPs were found to be toxic to HDMEC and hcMEC/D3 cells when assessed by the MTS test but were not toxic with the LDH test despite their increased intracellular concentration when assessed with TEM and with inductively coupled plasma and atomic emission spectroscopy (ICP-AES) [7].

In this study, we demonstrate the use of two label-free technologies to investigate the cytotoxicity and uptake of 14 nm and 20 nm citrate stabilized AuNPs in three cell lines. Through the use of cell impedance technology, potential interference of the nanoparticles with the toxicity test can be eliminated; whilst the use of dark-field imaging and hyperspectral imaging can confirm the presence of nanoparticles within the cells studied.

## Results

### Characterisation

**Figure 1:**
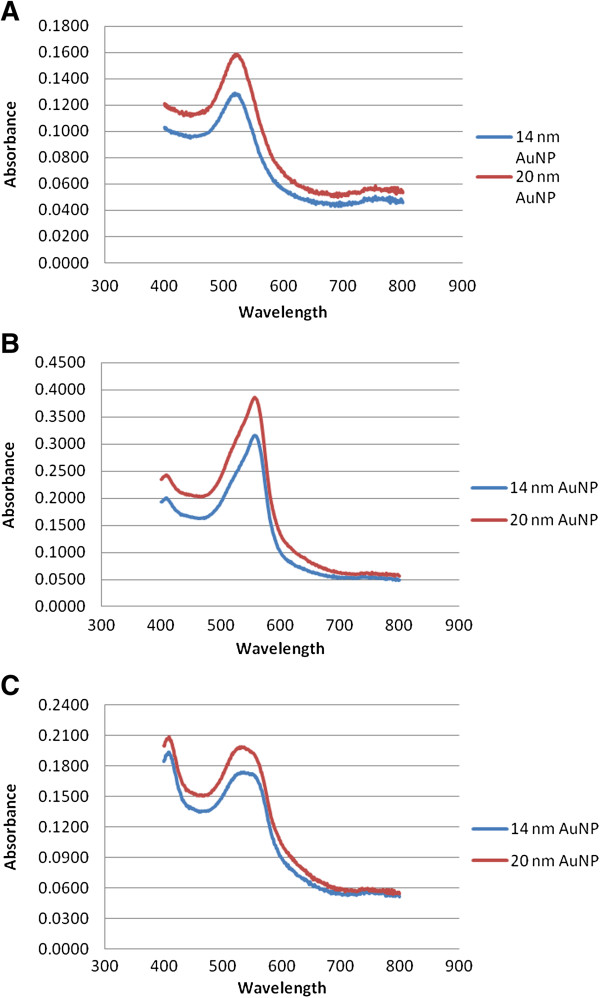
**Absorbance spectrum of the gold nanoparticles.** The UV–vis spectrum of 14 nm and 20 nm AuNPs at 1 nM in **A)** milli-Q water, **B)** in RPMI culture medium, and **C)** in Ham’s F12 culture medium.

**Figure 2:**
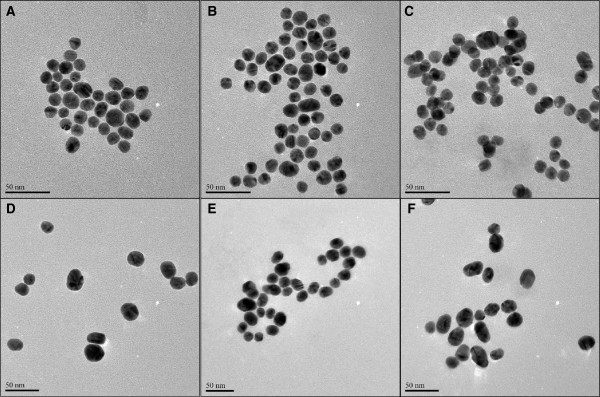
**Characterisation of the gold nanoparticles in milli-Q water and in culture medium using Transmission Electron Microscopy.** TEM images of 14 nm AuNPs in **(A)** water, **(B)** RPMI culture medium, and **(C)** Ham’s F12 culture medium; and 20 nm AuNPs in **(D)** water, **(E)** RPMI culture medium, and **(F)** Ham’s F-12 culture medium.

Table 1 shows the physicochemical properties of the AuNPs in milli-Q H_2_O. The surface charge of the particles, as described by their zeta (ζ) potential, are all negatively charged and of similar value. Table 2 shows the physicochemical properties of the AuNPs after centrifugation and resuspension in both RPMI and Ham’s-F12 culture medium. The pH of the RPMI and Ham’s-F12 culture medium at 37°C are pH 7.66 and 7.96 respectively. In all instances, the pH of the culture medium decreased slightly when the particles were resuspended therein; however the observed decreases were not biologically significant. The particles dispersed in Ham’s F12 culture medium showed a broader peak as compared to those in RPMI culture medium and water (Table 2 and Figure 1), owing to the difference in the refractive index of the medium and the final composition. AuNPs were further characterised with TEM and the representative images are shown in Figure 2. The TEM data demonstrates that the integrity of the AuNPs suspended in cell culture medium was maintained as compared to the AuNPs in water, with minimal signs of particle aggregation present. The AuNPs were primarily spherical in shape, with some ellipsoid nanoparticles present, probably due to synthetic conditions employed as factors such as salt concentration, pH, and temperature during synthesis have been shown to affect the polydispersity of AuNPs [18,19].

**Table 1:** Physicochemical properties of gold nanoparticles in milli-Q water

**Nanoparticle**	**Particle size determined by TEM**	**ζ-potential**	**Citrate concentration**	**UV–vis**	**Particles/ml (at 1 nM)**
14 nm AuNP	14 ± 2 nm	−33.5 mV	0.025%	520 nm	2.25 x 10^12^ nps/ml
20 nm AuNP	20 ± 2 nm	−37.9 mV	0.023%	524 nm	7.76 x 10^11^ nps/ml

**Table 2:** Physicochemical properties of gold nanoparticles suspended at 1 nM in culture medium

**Nanoparticle**	**ζ- Potential (mV)**		**pH at 37°C**		**Absorbance peak**		**Average particle size in nm**	
							**determined by TEM (Std Dev)**	
	**RPMI**	**Ham’s-F12**	**RPMI (pH 7.66)**	**Ham’s-F12 (pH 7.96)**	**RPMI**	**Ham-F12**	**RPMI**	**Ham-F12**
14 nm AuNP	−10.24	−11.40	7.61	7.84	557 nm	535 nm	17 nm (± 3 nm)	16 nm (± 3 nm)
20 nm AuNP	−12.06	−11.30	7.61	7.87	557 nm	532 nm	22 nm (± 4 nm)	24 nm (± 3 nm)

### Toxicity studies

### XTT, LDH, and ATP assays

**Figure 3:**
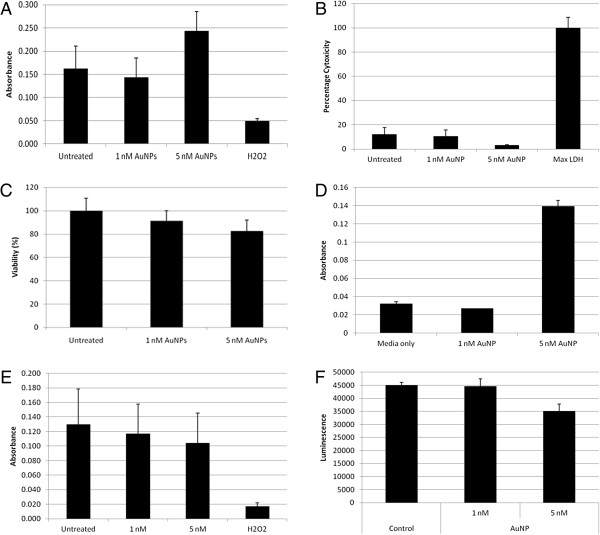
**Toxicity studies using conventional assays: XTT, LDH, and ATP assays. (A)** BEAS-2B cells were treated with either 1 nM or 5 nM of 14 nm AuNPs for 1 hour prior to toxicity testing using the *In Vitro* Toxicology Assay Kit (XTT assay). Absorbance measured at 450 nm and a reference wavelength of 600 nm. **(B)** BEAS-2B cells were treated with either 1 nM or 5 nM of 14 nm AuNPs for 1 hour prior to toxicity testing using the CytoTox-ONE™ assay (LDH assay). Lysis solution was used as a positive control for maximum LDH release and sample LDH is expressed as a percentage of this maximum release. Fluorescence measured at 560 nm excitation, 590 nm emission. **(C)** BEAS-2B cells were treated with either 1 nM or 5 nM of 14 nm AuNPs for 1 hour prior to toxicity testing using the CellTiter Glo assay (ATP assay). Luminescent signal was measured and viability is expressed as a percentage of the untreated control cells. **(D)** The absorbance of 1 nM and 5 nM AuNPs in culture medium and with unreduced XTT at 450 nm. **(E)** Absorbance values obtained when data from **(D)** was subtracted from **(A)**. **(F)** Luciferin substrate from the CellTiter Glo assay was incubated with ATP and 14 nm AuNPs to produce the luminescent product oxyluciferin prior to measurement of the luminescent signal.

Figure 3A shows the toxicity of the 14 nm AuNPs in BEAS-2B cells based on the XTT (2,3-bis-(2-methoxy-4-nitro-5-sulfophenyl)-2H-tetrazolium-5-carboxanilide) assay. In the figure the hydrogen peroxide positive control showed a decrease in absorbance, indicative of a decrease in cell viability. Based on the XTT assay, it can be deduced that at 1 nM the AuNPs exhibit only mild toxicity, however at 5 nM there is an increase in viability as compared to the untreated control cells. Figure 3B shows the cytotoxicity of the 14 nm AuNPs as determined by the CytoTox-ONE™ assay. The principle of this assay is the measurement of the release of LDH through the measurement of the fluorescence of the final assay product, resorufin. Cytotoxicity was calculated in accordance to the manufacturer’s instructions and is expressed as a percentage of maximum LDH release. From this assay data, the AuNPs are considered non-toxic to the cells under the same conditions as the XTT experiment. Figure 3C shows the toxicity of 14 nm AuNPs assessed by the CellTiter-Glo® Luminescent Cell Viability Assay which indicates the amount of ATP present. Cells were lysed to release ATP, followed by a luciferase reaction to produce a stable luminescent signal proportional to the ATP present. In this assay system, the manufacturer’s guidance manual states that cell washing and the removal of culture medium is not required. From the results presented in this figure, it can be concluded that AuNPs were toxic in a concentration dependent manner.

Contradictory cytotoxicity results obtained between the XTT-, LDH-, and ATP-based assays suggests possible interference of tested AuNPs with these three assay systems. Indeed, such interference could be confirmed by the concentration-dependent increase in the absorbance by AuNPs at a wavelength of 450 nm in the absence of cells but in the presence of unreduced XTT (Figure 3D). Subsequently, the absorbance of particle-containing medium controls, as seen in Figure 3D, was subtracted from the XTT viability data, shown in Figure 3A. From this amended data (Figure 3E), a conclusion can be made that dose-dependent toxicity is produced relative to the untreated cells.

However, no meaningful interference on fluorescence or luminescence measurements were observed when particles alone, resuspended in medium, were included in the LDH and ATP assays (results not shown). However, when a further experiment was conducted to investigate the effects of the AuNPs on the reaction that occurs during the ATP-based assay, namely the conversion of luciferin substrate to luminescent oxyluciferin in the presence of ATP, it can be seen that with an increase in AuNP concentration, a decrease in luminescent signal is observed (Figure 3F), suggesting that the AuNPs are interfering with the conversion of luciferin to oxyluciferin at high concentrations. If a comparison of results is made between Figure 3C and 3F, it is possible that the observed decrease in viability at 5 nM AuNPs in Figure 3C could be caused by interference of the AuNP with the assay reaction.

The accepted norm for the application of these assay systems is the inclusion of medium controls in the experimental setup with no attention to the optical properties of the particles themselves or the particles’ interactions with the assay reagents. It can therefore be concluded that such interference by AuNPs with detection of the assay end-product by absorbance cannot be ignored for the XTT assay system. However, within the ATP assay, the AuNPs do not produce a luminescent signal themselves, but rather interfere with the conversion of the substrate to a luminescent product.

### Dynamic monitoring of cytotoxic response using RTCA cell impedance system

**Figure 4:**
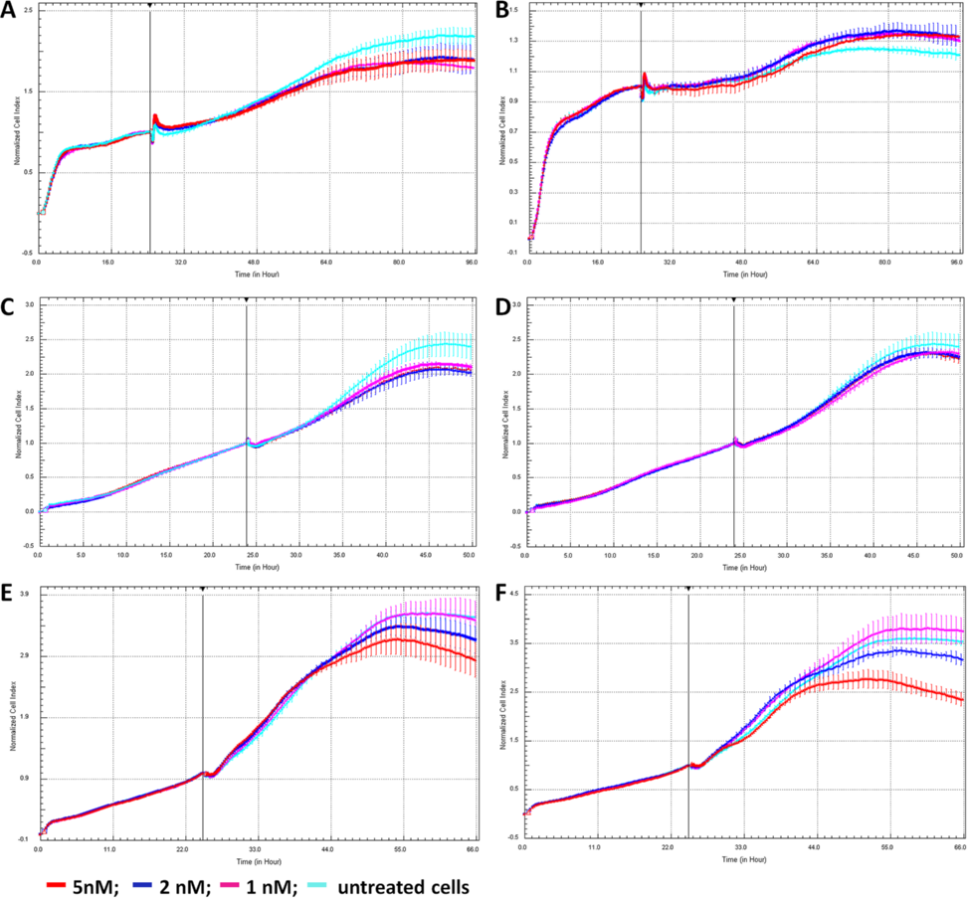
**Normalised cell index of cells treated with AuNPs.** BEAS-2B cells **(A** and **B)**, HEK 293 cells **(C** and **D)**, and CHO cells **(E** and **F)** were treated with 14 nm AuNPs (left panel) and 20 nm AuNPs (right panel). BEAS-2B, HEK 293, and CHO cells were seeded and allowed to recover for 24 hours prior to treatment for 72 hours, 36 hours, or 42 hours, respectively. The slope of the curves was calculated and t-test conducted to determine differences between treated and untreated control cells. In **(A)** and **(C)**, all treatments were statistically different from untreated control (p < 0.05) whilst no significant differences were observed in **(B)** and **(D)**. In **(E)**, 2 nM (p < 0.05) and 5 nM (p < 0.01) were statistically different from untreated control; whilst in **(F)** both 2 nM and 5nM treatments were statistically different from the untreated control at p < 0.01; n ≥ 3.

Contradictory results with the XTT, LDH and ATP assay systems and the subsequent illustration that AuNPs interfere in the absorbance readings of the XTT assay as well as the ATP assay, has necessitated the use of an alternate system that does not rely on optical detection of the final products. Impedance technology such as the xCELLigence Real-TimeCell Analyzer (RTCA) system assesses the cell index and therefore eliminates problems with interference of the AuNPs with determination of cell viability. The normalised cell index is indicative of the level of adhesion and therefore associated with the viability of the cells. AuNPs alone in culture medium showed no effect on cell index whereas treating BEAS-2B cells with 500 μM hydrogen peroxide used as a positive control for cell death caused a decrease in cell index (See Additional file 1: Figure S1). Figure 4 illustrate the normalised cell index over time of the BEAS-2B (Figure 4A and B), HEK 293 (Figure 4C and D), and the CHO cells (Figure 4E and F) when treated with the 14 nm and 20 nm AuNPs. In all cases, cells were treated for 24 hours after seeding and the cell index was normalised at the point of treatment. Each cell line had its own unique growth curve and treatment was carried out whilst the cells were in the early stages of log growth phase. The slope of the curves, a term describing the changing rate of the CI within a given time period, was calculated between the point of treatment and point of confluence, and any significant differences between the untreated and treated slopes are indicated in figure legend. The cells treated with 14 nm AuNPs showed significant differences from the untreated controls within the BEAS-2B and the HEK 293 experiments (Figure 4A and C). In both instances, the treated cells showed a similar growth trend as the untreated cells and no dose-dependent response was observed. However, both the 14 nm and 20 nm AuNPs showed significant differences and an apparent dose–response to the CHO cells (Figure 4E and F), and the 20 nm AuNPs showed greater toxicity based on the greatest decrease of CI.

### Intracellular uptake using hyperspectral imaging microscopy

**Figure 5:**
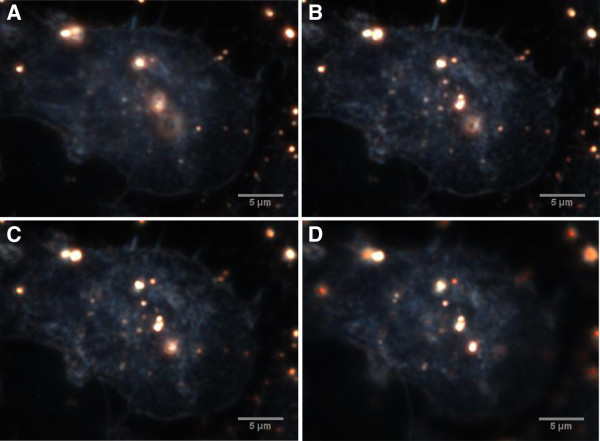
**Dark-field image at 100x magnification of BEAS-2B cells incubated with 14 nm AuNPs for 1 hr.** Cells were washed, fixed, and immobilised onto slides prior to image capture using the CytoViva system. Four focal planes, **(A)** through to **(D)**, are observed as the focus of the microscope moves through the cell.

The CytoViva Hyperspectral Imaging (HSI) System provided dark-field images of the cells following their incubation with AuNPs and fixation. To observe the internalisation of the particles, it was possible to focus the microscope on various focal planes throughout the fixed cells. Figure 5 shows a single BEAS-2B cell that has been incubated with 14 nm AuNPs at 1 nM for an hour. The image illustrates 4 focal planes within the cell. In (A) the particles are out of focus, but as the focus of the microscope moves through (B) to (D), one is able to observe various particles within the outline of the cell which come into and then go out of focus, thereby confirming that the AuNPs have been internalised. This approach has been used to confirm internalisation of TiO_2_ NPs in fibroblasts [20], and silver NPs in nematodes [21].

**Figure 6:**
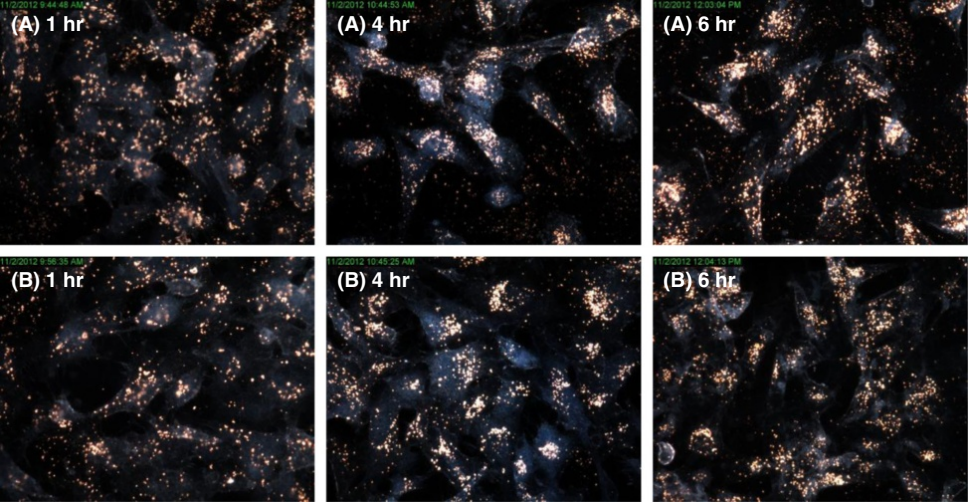
**Dark-field images of BEAS-2B cells incubated with AuNPs.** Dark-field images were captured at 60x magnification of BEAS-2B cells incubated with either **(A)** 14 nm or **(B)** 20 nm AuNPs for 1 hr, 4 hrs, or 6 hrs.

**Figure 7:**
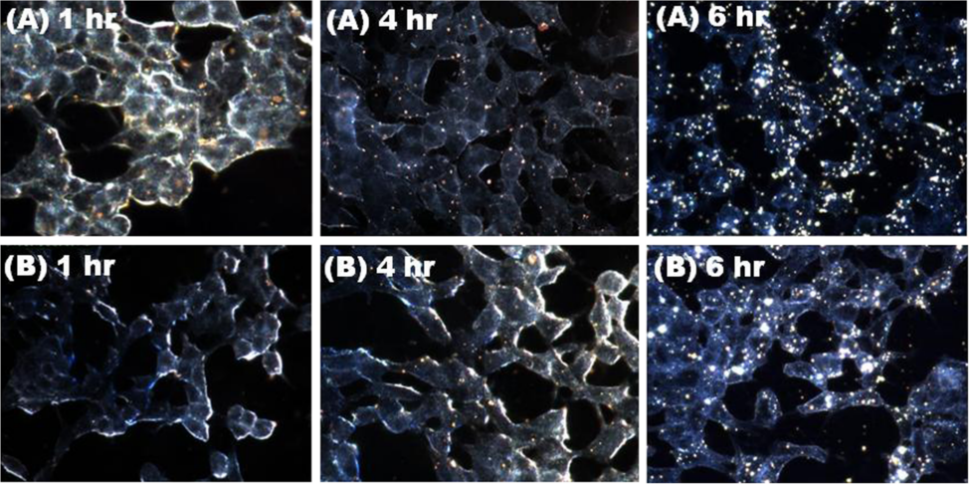
**Dark-field images of HEK 293 cells incubated with AuNPs.** Dark-field images were captured at 60x magnification of HEK 293 cells incubated with either **(A)** 14 nm or **(B)** 20 nm AuNPs for 1 hr, 4 hrs, or 6 hrs.

**Figure 8:**
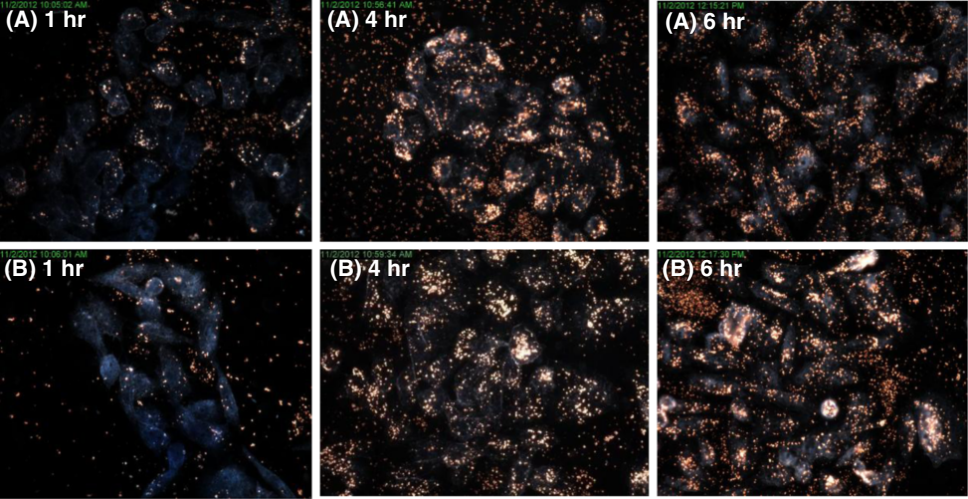
**Dark-field images of CHO cells incubated with AuNPs.** Dark-field images were captured at 60x magnification of CHO cells incubated with either **(A)** 14 nm or **(B)** 20 nm AuNPs for 1 hr, 4 hrs, or 6 hrs.

Figure 6 confirms the uptake of the AuNPs in BEAS-2B cells. The 14 nm (Figure 6A) and 20 nm (Figure 6B) showed high levels of uptake, even after an hour of incubation. The particles seemed to accumulate and form aggregates within the cells. Figure 7 demonstrates the uptake of the AuNPs into HEK 293 cells. The cells treated with 14 nm AuNPs (Figure 7A) had cellular uptake after an hour, whereas those treated with 20 nm AuNPs (Figure 7B) had minimal cellular uptake. There was some accumulation of all nanoparticles in the cells after 6 hours, although this uptake was not as high as in BEAS-2B cells. Figure 8 presents the uptake of the 14 nm and 20 nm AuNPs in CHO cells. Minimal AuNP uptake was observed in the CHO cells after an hour, however an increase and high accumulation of the nanoparticles was observed for the 14 nm (Figure 8A) and 20 nm (Figure 8B) AuNPs after 4 and 6 hours with the 20 nm AuNPs cellular uptake being higher than that of the 14 nm AuNPs.

**Figure 9:**
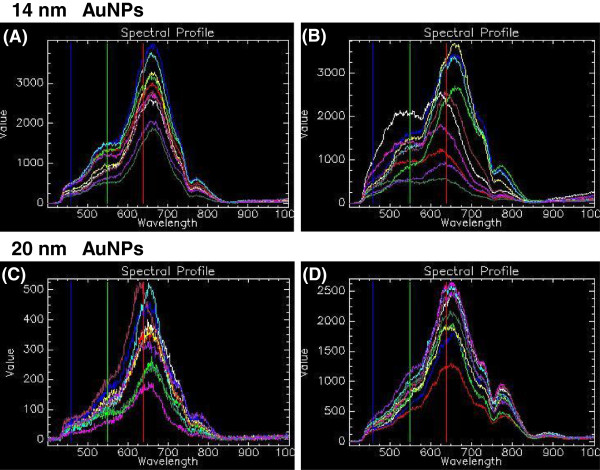
**Spectral profiles of 14 nm and 20 nm AuNPs.** Ten spectral profiles of randomly selected AuNPs that appeared visually to be either **(A** and **C)** in single suspension, or **(B** and **D)** aggregated nanoparticles. Each coloured line represents the spectrum from a single pixel.

Figure 9 represents the spectral profiles of 14 nm and 20 nm AuNPs. Nanoparticle solution was spread on a slide and allowed to dry prior to HSI. Ten randomly selected spectra were obtained from nanoparticles that appeared to be either singularly dispersed (A and C) or had formed larger aggregates (B and D) on the slide. Each coloured line represents the spectrum from a single pixel, and these collections of representative spectra form a known spectral library of the nanoparticles. In these figures, it can be seen that individually dispersed AuNPs of the two sizes tend to have homogenous curves within the sample, as represented in Figure 9A and C for 14 nm and 20 nm nanoparticles, respectively. The AuNPs at both sizes tend to have a predominant single peak at approximately 670 nm. Figure 9B and D illustrate a range of spectra for aggregated 14 nm and 20 nm AuNPs, respectively. The non-uniformity of these spectra may probably be due to the different sizes of the agglomerates in the sample. In addition, the spectral profiles of the aggregated AuNPs tend to have broader peaks than their singularly dispersed counterparts as well as the appearance of a second peak at approximately 800 nm. When the well-known statistical algorithm Principal Component Analysis (PCA) was performed on these spectra to identify the principal components (PCs) that are responsible for the spectral variation between the different sized nanoparticles, the score plot obtained could not discriminate between the different sizes (See Additional file 1: Figure S2). Subsequently, these spectra may be used to confirm the presence of gold nanoparticles but may not be used to discriminate between particle sizes.

**Figure 10:**
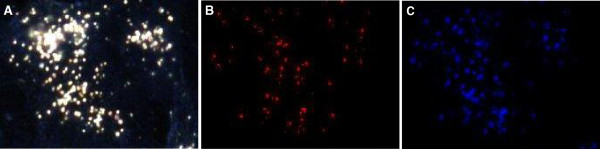
**Representative images of the SAM analyses. (A)** HSI scan of BEAS-2B cells treated with 1 nM 14 nm AuNPs for 4 hours; **(B)** SAM image indicating pixels of **(A)** that matched the spectral library of 14 nm singularly dispersed nanoparticles; **(C)** SAM image indicating pixels of **(A)** that matched the spectral library of the 14 nm aggregated nanoparticles.

Spectral Angle Mapper (SAM) is a process for the identification of a known material in an image acquired using hyperspectral imaging. The process is performed by comparing the known spectra for a material to unknown spectra and identifying pixels in the unknown image that map the known spectra, irrespective of the light intensity. Using this process on scans of the cells incubated with the AuNPs, a representative output was obtained (Figure 10). In this figure the HSI scan of BEAS-2B cells incubated with 1 nM 14 nm AuNPs for 4 hours represents an “unknown” spectral image (Figure 10A). The known spectral library of the singularly dispersed 14 nm AuNPs was mapped against this image (Figure 10B), and the spectral library of aggregated 14 nm AuNPs was also mapped (Figure 10C). Both the aggregated and singularly dispersed spectral libraries mapped onto the images of the cells, thereby verifying the presence of gold nanoparticles in the cells. However it can be seen that the aggregated AuNPs matched more pixels within the cells than the singularly dispersed AuNPs indicating that there is a better match with the aggregated spectral library than the singularly dispersed spectral library. Therefore, the presence of aggregated rather than singularly dispersed gold nanoparticles could be assumed. This latter observation made when using SAM concurs with visual observations made from the spectral profiles of AuNPs in the absence of cells (Figure 9) and those from inside the cells (See Additional file 1: Figure S3 and S4).

## Discussion

Many commercially available toxicity kits rely on the optical properties of the reaction product as the basis of the detection assay. However, AuNPs absorb light in the visible region and also are able to potentially adsorb molecules such as indicator dyes [22]. Interference would therefore be expected when using the XTT assay. Although the assay-data presented with XTT on the toxicity of AuNPs agree with those presented earlier by other investigators using MTT [7], the lack of toxicity of these nanoparticles using LDH is in contrast with those presented by these same authors. This could be due to the fact that kits used by these authors were absorbance based, where the absorbance of the final product was measured at 490 nm; whereas in the study presented herein, the detection of the release of LDH was fluorescence-based. Interference was also observed with the ATP bioluminescence assay system; however this was not due to the optical properties of the AuNPs, but rather interference with the production of the measured end-product. It is therefore possible that the contradictory data on the toxicity of AuNPs may be as a result of interference of these AuNPs with the toxicity assay substrate and also their subsequent assay products.

Similar observations have been reported on the interference of nanoparticles other than AuNPs when using toxicity test systems such as MTT (similar to XTT), 2′,7′-Dichlorofluorescein (DCF), and IL-8 ELISA assays [23]. In an earlier review, while discussing different *in vitro* methodologies used for nanoparticle toxicity assessment, these authors have summarized nanoparticle characteristics such as high adsorption capacity, surface charge, and catalytic activity as potential causes of the interference of nanoparticles with these toxicity assays [24]. Kroll *et al.*, [23] suggested an approach to prevent interference of nanoparticles with *in vitro* toxicity assays that involves altering assay protocols or lowering particle concentrations. However, the authors did observe that interference was assay- and particle-specific, and recommended that each *in vitro* methodology be evaluated for each individual nanoparticle. Although effective, this validation is time-consuming and possibly expensive, especially if the results of the validation show that the interference by nanoparticles cannot be overcome. We therefore suggest the use of cell impedance to ascertain the cytotoxicity of nanoparticles especially with those that have plasmon effects such as gold and silver nanoparticles.

The xCELLigence RTCA technology measures impedance changes caused by the gradual increase of electrode surface occupation by cells represented as a Cell Index (CI) which reflects the state of the ionic environment at the electrode/solution interface. This impedance is influenced by the presence of cells cultured on plates on top of these electrodes, and therefore CI values were used to monitor cell viability where cell adhesion was considered as the cell viability end-point [25,26]. Cell-based label-free technologies such as the xCELLigence RTCA system have been implemented as part of the preclinical drug development process [27-29] and also for the evaluation of the cytotoxicity of a variety of dissolved chemicals [30-33]. This technology was also used to assess the cytotoxicity of a variety of nanoparticles including quartz [34], cobalt ferrite [35], gold nanoparticles [36], quantum dots and gold nanorods [37], ZnO, CuO, and TiO_2_[38] and other inorganic nanoparticles [39].

Using this impedance technology, it was possible to assess the toxicity of 14 nm and 20 nm AuNPs on three different cell lines. It was found that the decrease of cell index was slight for both 14 nm and 20 nm AuNPs, but only significant for the 14 nm AuNPs, indicating minimal toxicity to the BEAS-2B cells (Figure 4A and B). Similar findings were obtained with the HEK 293 cells (Figure 4C and D). The most sensitive cells to the AuNPs were the CHO cells with 20 nm AuNPs having the highest toxicity (Figure 4E and F).

It is important to note that our results, generated with the RTCA technology, on the toxicity of 14 nm AuNPs did not correlate well with those generated with the conventionally used XTT- or ATP-based toxicity assays. Positive correlation was however reported in the literature. For example, a correlation was found in the toxicity of drugs when assessed by either neutral red uptake assay or the RTCA [29], or in the cytotoxicity of sodium arsenite, cadmium chloride and *cis*-platinum assessed by MTT and RTCA [33], or in the immunocytotoxicity of baboon sera on pig cells with the MTT and RTCA [40] or cell toxicity assay using the Sulforhodamine B for staining proteins and measuring absorbance at a wavelength of 565 nm in correlation to RTCA [41]. Such correlation was even observed between impedance-based technology and the MTS assay for the assessment of CTAB-capped gold nanoparticles of difference shapes [36,37]. The difference between those reported in the literature and those observed in the present experiments is that we have found that the materials tested have shown absorbance or luminescence (Figure 3) and therefore have interfered in the assessment of the final products produced from these test systems and subsequently no such correlation could be observed between our data generated by the conventional assay systems and that of the RTCA. Although the lack of correlation observed between the XTT assay system and those generated with impedance technology may be in contrast to those reported earlier with the MTS assay system [36,37], the explanation would be that the interference caused in these assays results in apparent increased toxicity, irrespective of actual toxicity, whilst the impedance technology presents a more accurate representation of whether something is toxic or non-toxic.

In the present study, CytoViva’s hyperspectral imaging technology was used to assess intracellular uptake by three different cell lines of two sizes of AuNPs; namely 14 and 20 nm. This technology was previously implemented to determine the potential distribution of multi-walled carbon nanotubes (MWCNT) in different regions and target cells in the lung [42], for the assessment of a panel of metal nanoparticle catalysts for their antifungal activities against *Candida albicans*[43], and for the assessment of internalization of silver nanoparticles and subsequent cellular changes in U937 cells in relation to toxicity [44]. Via the hyperspectral imaging technology, we were able to assess intracellular uptake of the 14 nm and 20 nm AuNPs by BEAS-2B cells at high levels and with minimal toxicity detected, with the observation that these particles were aggregated once internalized by these cells. Similarly, the uptake of these two different sizes of AuNPs by the HEK 293 cells was time dependent and the 20 nm AuNPs did not produce a toxic response. Finally, the uptake of AuNPs by the CHO cells was size dependent where the 14 nm and the 20 nm AuNPs could enter cells in a time-dependent manner with higher toxicity found with the 20 nm AuNPs. Differences in uptake by HeLa, A549, and 1321N1 cell lines have previously been shown for 40 nm and 200 nm polystyrene nanoparticles [45].

As both AuNPs tested were negatively charged, any differences in uptake within a cell system are probably due to size differences. Resuspension of AuNPs in RPMI or Ham’s-F12 medium did increase the ζ-potential and subsequently may have increased the tendency for the nanoparticles to aggregate. This was confirmed with the differences in the hydrodynamic size of the nanoparticles measured in the different cell culture media using DLS (see Additional file 1: Table S1 and Figure S5). It is also worthwhile mentioning that the individual composition of the different cell culture media could affect protein corona formation, which, in turn, would also affect uptake and possibly the toxicity of the nanoparticles [46].

It was possible to show, with CytoViva measurements, that aggregation of the nanoparticles occurred after the uptake by the three different cell types. This aggregation could be visually observed due to the high signal-to-noise dark-field images, and confirmed through SAM analyses. As for the mechanism of internalization of the negatively charged AuNPs within the three different cell types, it has been previously postulated that uptake of negatively charged AuNPs may be mediated through nonspecific adsorption of serum proteins onto the gold surface which may aid nanoparticle entry via the mechanism of receptor mediated endocytosis [13]. Further studies will therefore be conducted to elucidate mechanisms of AuNP uptake, within various cell lines, using the CytoViva system.

This study demonstrates the use of two label-free technologies, namely cell impedance and hyperspectral imaging, as desirable systems for use in the assessment of toxicity and uptake of AuNPs in adherent cell lines. These systems have assisted us in circumventing the contradictory results observed with the use of the traditional XTT, LDH and ATP assays for the cytotoxicity of AuNPs.

## Conclusions

The two methodologies presented herein have shown to be effective in the analysis of cytotoxicity and uptake of AuNPs. An advantage of these techniques is that both are label-free and thereby eliminate potential interference with detection. Interference was overcome with the use of cell impedance to measure cytotoxicity; and the CytoViva system provided dark field images of AuNP internalisation and thus allowed the analysis of the spectral profiles of the nanoparticles without interference.

## Methods

### Synthesis of AuNPs

Tetrachloroaurate (HAuCl_4_.3H_2_O) and trisodium citrate (Na_3_C_6_H_5_O_7_.2H_2_O) were purchased from Sigma Aldrich and used without further purification. The 14 nm and 20 nm AuNPs were prepared with sodium citrate as the reducing agent according to literature methods [47,48]. In brief, the 14 nm gold nanoparticles were prepared by adding trisodium citrate aqueous solution (10 mL, 17 mM) into 180 mL (0.3 mM) of boiling HAuCl_4_.3H_2_O aqueous solution. The mixture was boiled under reflux for 15 minutes and the resultant suspension allowed to cool to room temperature. The deep red citrate-capped gold nanoparticle suspension was further stirred overnight followed by filtration using 0.25 μm syringe sterile filter before use. The 20 nm gold nanoparticles were prepared in a similar way, but with 8.8 mL of tri-sodium citrate added into 180 mL of 0.3 mM HAuCl_4_.3H_2_O aqueous solution. The synthesis was performed under sterile conditions.

### The physicochemical properties of the synthesized citrate-capped gold nanoparticle

The physicochemical properties of the AuNPs in milli-Q water and in two cell culture medium were determined. For the stability of AuNPs in culture medium, the nanoparticles suspended in milli-Q H_2_O, were centrifuged at 13 000 x g for 30 min and re-suspended in RPMI culture medium or Ham’s culture medium and further characterized. The absorbance spectrum of the AuNPs was determined on the Thermo Scientific Multiskan GO spectrophotometer. Zeta potential (ζ-potential) measurements were performed using Malvern Instruments’ Zetasizer Nano ZS while pH measurements were performed at 37°C using the CyberScan pH 6500 instrument. TEM, operating at 120 kV using JOEL-JEM 1010, was used for size measurement experiments where colloidal samples were deposited, as droplets, on the carbon coated copper grids and allowed to dry prior to analysis. A minimum of 50 nanoparticles were measured using ImageJ 1.46r (National Institutes of Health, USA) to obtain an average particle size.

### Cell cultures

Three cell lines were used in this study, namely the bronchial epithelial cell line BEAS-2B, the Chinese hamster ovary CHO cell line, and the human embryonic kidney cell line HEK 293. Cell lines were routinely cultured under standard culturing conditions (37°C, 5% CO_2_ in a humidified environment). BEAS-2B cells and HEK 293 cells were cultured in RPMI-1640 medium with L-glutamine, 10% heat-inactivated foetal bovine serum (FBS) and 1% Penicillin/Streptomycin, hereafter referred to as RPMI culture medium. CHO cells were cultured in Ham’s-F12 with L-glutamine, 10% heat-inactivated FBS and 1% Penicillin/Streptomycin, hereafter referred to as Ham’s F12 culture medium. After reaching sub-confluency, the monolayer was washed with phosphate buffered saline (PBS) and harvested by a brief incubation with a trypsin/EDTA solution. The cells were resuspended in growth medium and viability determined by the trypan blue cell viability assay. Cell culture reagents were purchased from Lonza, Belgium.

### Toxicity

#### Assessment of cytotoxicity using conventional assays

BEAS-2B cells were seeded at 1 × 10^4^ cells/well in a 96-well plate and allowed to proliferate for 24 hours before AuNP treatment. RPMI culture medium without phenol red was used. Untreated control wells contained medium as described. Cells were treated with 1 or 5 nM of 14 nm gold nanoparticles for an hour. Cell viability was then assessed using both the *In Vitro* Toxicology Assay Kit, XTT based (Sigma-Aldrich), the CytoTox-ONE™ Homogeneous Membrane Integrity Assay (Promega), and the ATP CellTiter Glo assay (Promega).

a. XTT assay: Positive control wells received 500 μM hydrogen peroxide (Merck). XTT was reconstituted with 1% PMS and as per the manufacturer’s instructions, and added to each well to a final volume of 20% per well. The plate was incubated for 2 hours and the absorbance of which is read at 450 nm. To test for interference of the AuNPs with the optical readout, the absorbance of 1 nM and 5 nM AuNPs in culture medium with unreduced XTT was measured.

b. CytoTox-ONE™ assay: This assay is based on the measurement of the release of lactate dehydrogenase (LDH). Lysis solution provided with the kit was used to generate maximum LDH Release. The CytoTox-ONE™ Reagent was added to each well in a 1:1 ratio. The plate was incubated at 22°C for 10 mins. Stop solution was added and the plate was shaken for 10 secs. LDH released into the culture medium is measured with a 10-minute coupled enzymatic assay that results in the conversion of resazurin into resorufin. Fluorescence of resorufin was then measured at 560 nm excitation, 590 nm emission.

c. The CellTiter-Glo® Luminescent Cell Viability Assay: This is based on the quantitation of ATP, which signals the presence of metabolically active cells. The CellTiter-Glo® Reagent was added directly to cells in multiwell plates. The plate was incubated at room temperature for 10 minutes, to lyse the cells, for the release of the ATP. Luciferin is catalyzed by luciferase in the presence of Mg^2+^, ATP and molecular oxygen to generate a stable luminescent signal. To test for interference of AuNPs with the conversion of substrate to product, the CellTiter-Glo® Reagent was incubated with 1.5 μM ATP as a co-factor and AuNPs.

#### Assessment of cytotoxicity using cell impedance

Cytotoxicity studies were conducted using the xCELLigence RTCA single plate (SP) instrument from ACEA Biosciences with RTCA software (version 1.2). Cell Index (CI) is a measure of the electrode impedance which reflects the state of the ionic environment at the electrode/solution interface. This impedance is influenced by the presence of cells cultured on plates on top of these electrodes, and therefore the CI values are used to monitor cell viability.

BEAS-2B, HEK 293, and CHO cells were seeded at 1 × 10^4^ cells/well, 5 × 10^4^ cells/well, and 3 × 10^3^ cells/well respectively in a 96-well E-Plate. Cells were placed in the RTCA station and allowed to proliferate for 24 hours for all cells prior to treatment, during which time a scan was acquired every 15 minutes. The cells were then treated with the 14 nm or 20 nm AuNPs at final concentrations of 1 nM, 2 nM, and 5 nM. Scans were acquired every minute for 2 hours, and then every 15 minutes for the remainder of the experiments.

For statistical analysis, within each experiment, CI of each curve was normalized at the point of treatment. The slope for each curve of the normalized CI for the time period between treatment point and the highest CI value, i.e. confluency, of the untreated cells was calculated. The calculated slopes were exported and a t-test was conducted using STATISTICA version 10 (StatSoft, Inc.) to investigate if the differences between treated and untreated slopes were significant. Differences were considered statistically significant if p < 0.05.

### Uptake

BEAS 2B, HEK 293, and CHO cells were seeded at 3 × 10^4^ cells/cm^2^, 7.6 × 10^4^ cells/cm^2^, and 1.6 × 10^4^ cells/cm^2^, respectively, in 8-well Millicell EZ-slides (Millipore, Ireland). Cells were allowed to proliferate for 24 hours prior to treatment at 1 nM for 1, 4, or 6 hours. Following treatment, cells were washed three times with relevant culture medium, followed by three washes with Dulbecco’s Phosphate Buffered Saline (DPBS). Cells were fixed at 4°C with 4% formalin in Tris/HCl buffer for 15 mins. Slides were washed once with DPBS and air-dried. Coverslips were immobilised onto the slides with Kaiser’s gelatine.

Dark-field images were captured at 60x magnification using the CytoViva 150 Unit integrated onto the Olympus BX43 microscope. Images were acquired using a Dagexcel X16 camera and the associated software. Hyperspectral Imaging (HSI) was performed at 60x magnification using the HSI System 1.1 and ENVI software. In addition to the scans of the cells treated with gold nanoparticles as described, scans were also performed on the AuNPs alone. For this analysis, a drop of AuNP solution was placed on a microscope slide, spread out and allowed to dry. A coverslip was placed on the slide prior to the acquisition of an HSI scan at 60x magnification. Spectral libraries were collected by selecting the spectra of particles that appear to either be singularly dispersed or had visually aggregated to form larger particles. The image classification algorithm SAM (spectral angle mapper) was conducted using the ENVI software to map the spectral libraries onto the scans of the cells incubated with particles. A threshold of 0.08 maximum acceptable angles (radians) between the known and unknown spectra was applied. In addition, these spectral libraries were visually compared to those of the AuNPs found within the cells. Principal Component Analysis (PCA) was performed (see Additional file 1).

## Abbreviations

ATP: Adenosine triphosphate; AuNP: Gold nanoparticle; CI: Cell index; DCF: Dichlorofluorescein; FBS: Foetal bovine serum; hcMEC/D3: Human cerebral microvascular endothelial (hcMEC/D3) cells; HDMEC: Human dermal microvascular endothelial (HDMEC) cells; HSI: Hyperspectral imaging; ICP-AES: Inductively coupled plasma and atomic emission spectroscopy; LDH: Lactate dehydrogenase; MTS: 3-(4,5-Dimethylthiazole-2-yl)-5-(3-carboxymethoxyphenyl)-2-(4-sulfophenyl)-2H-tetrazoliumin; MTT: 3-(4,5-dimethylthiazol-2-yl)-2,5-diphenyltetrazolium bromide; NPs: Nanoparticles; PCA: Principal component analysis; RTCA: Real-time cell analyzer; SAM: Spectral angle mapper; SEM: Scanning electron microscopy; TEM: Transmission electron microscopy; MWCNT: Multi-walled carbon nanotubes; PBS: Phosphate buffered saline; XTT: 2,3-bis-(2-methoxy-4-nitro-5-sulfophenyl)-2H-tetrazolium-5-carboxanilide.

## Competing interests

The authors declare that they have no competing interests.

## Authors’ contributions

AuNPs were synthesized by DTR, FKK, and AS. Characterization of the AuNPs was done by DTR, FKK, KB and MV. Interference work was performed by CA, KB, LK and MV. xCELLigence and CytoViva work was performed by NT and MV. Statistical analyses were done by MV. MV, RT, and MG were involved in the inception and planning of the project and in the preparation of the manuscript. All authors read and approved the final manuscript.

## Supplementary Material

Additional file 1: Figure S1 Cell index of BEAS-2B cells showing toxicity of 500 μM hydrogen peroxide and effect of 5 nM 20 nm AuNPs in medium on cell index. **Figure S2**. PCA analysis of 14 nm and 20 nm AuNP spectra. **Figure S3**. Spectral profiles of 14 nm AuNPs following cellular uptake. **Figure S4**. Spectral profiles of 20 nm AuNPs following cellular uptake. **Table S1.** Average hydrodynamic size of 14 nm and 20 nm AuNPs in culture medium as determined by DLS. **Figure S5**. Representative image of the bimodal distribution obtained from DLS. Click here for file
