# Supplementary material for: Label-free in vitro toxicity and uptake assessment of citrate stabilised gold nanoparticles in three cell lines
Source: Part Fibre Toxicol. 2013 Oct 9;10:50. doi: 10.1186/1743-8977-10-50 (PMC3853235; doi:10.1186/1743-8977-10-50)
Supplement: Additional file 1: Figure S1 — Cell index of BEAS-2B cells showing toxicity of 500 μM hydrogen peroxide and effect of 5 nM 20 nm AuNPs in medium on cell index. Figure S2. PCA analysis of 14 nm and 20 nm AuNP spectra. Figure S3. Spectral profiles of 14 nm AuNPs following cellular uptake. Figure S4. Spectral profiles of 20 nm AuNPs following cellular uptake. Table S1. Average hydrodynamic size of 14 nm and 20 nm AuNPs in culture medium as determined by DLS. Figure S5. Representative image of the bimodal distribution obtained from DLS. [file 1743-8977-10-50-S1.pdf]

Label-free in vitro toxicity and uptake assessment of citrate stabilised gold nanoparticles in three cell lines

Melissa A Vetten, Nonhlanhla Tlotleng, Delia Tanner Rascher, Amanda Skepu, Frankline K Keter, Kailen Boodhia, Leigh-Anne Koekemoer, Charlene Andraos, Robert Tshikhudo and Mary Gulumian

Content:

1. Supplementary Information on RTCA Cell Impedance System
2. Principal Component Analysis (PCA) on AuNP spectra
3. Supplementary Information on Spectral Profiles
4. DLS on NPs in culture media

1. Supplementary Information on RTCA Cell Impedance System

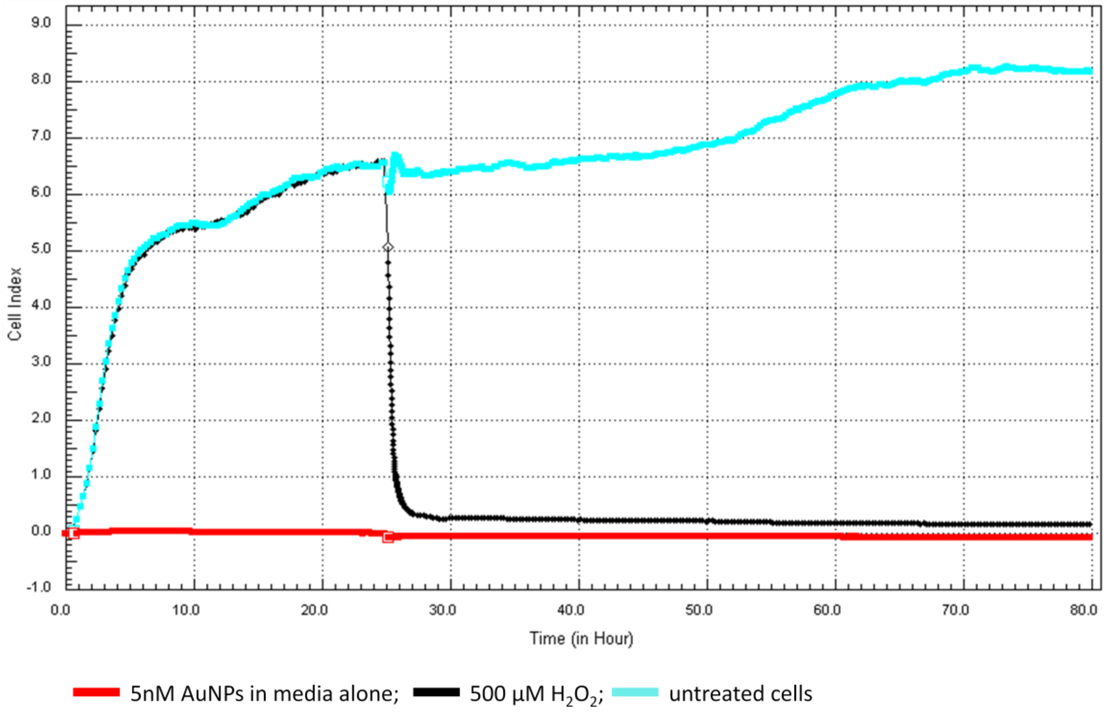

Figure S1. Cell index of BEAS-2B cells showing toxicity of 500 µM hydrogen peroxide and effect of 5 nM 20 nm AuNPs in medium on cell index.

BEAS-2B cells were either untreated or treated with 500 µM hydrogen peroxide as a positive control for cell death. The cell index of 5 nM 20 nm AuNPs in medium is shown in red. 20 nm AuNPs were added to wells containing medium at approximately 24 hours, resulting in negligible changes in the cell index. A similar trend was observed with 14 nm AuNPs.

2. Principal Component Analysis (PCA) on AuNP spectra

Image processing techniques such as principal component analysis are widely used for data dimensionality reduction and feature extraction [1-2]; therefore PCA was run on the spectra of singularly dispersed and aggregated 14 nm and 20 nm AuNPs.

Methods

A drop of either 14nm or 20 nm AuNP solutions were placed on a microscope slide, spread out and allowed to dry. A coverslip was placed on the slide prior to the acquisition of an HSI scan at 60x magnification. A minimum of 20 spectra were randomly selected from nanoparticles that appeared to be either singularly dispersed or had formed larger aggregates on the slide. Principal Component Analysis (PCA) was conducted on the spectra, including all wavelengths acquired, using Statistica version 12.

Results

The PCA score plot of Factor 1 vs Factor 2 is shown in Figure S2. The first two factors identified by PCA account for over 90% of the variance observed. It can be seen in this figure that the points representing singularly dispersed AuNPs are more closely orientated, whilst the points for the aggregated AuNPs are widely dispersed and no distinct clusters can be identified. This suggests that there is not sufficient difference between the spectra of the difference AuNPS for PCA to discriminate between them.

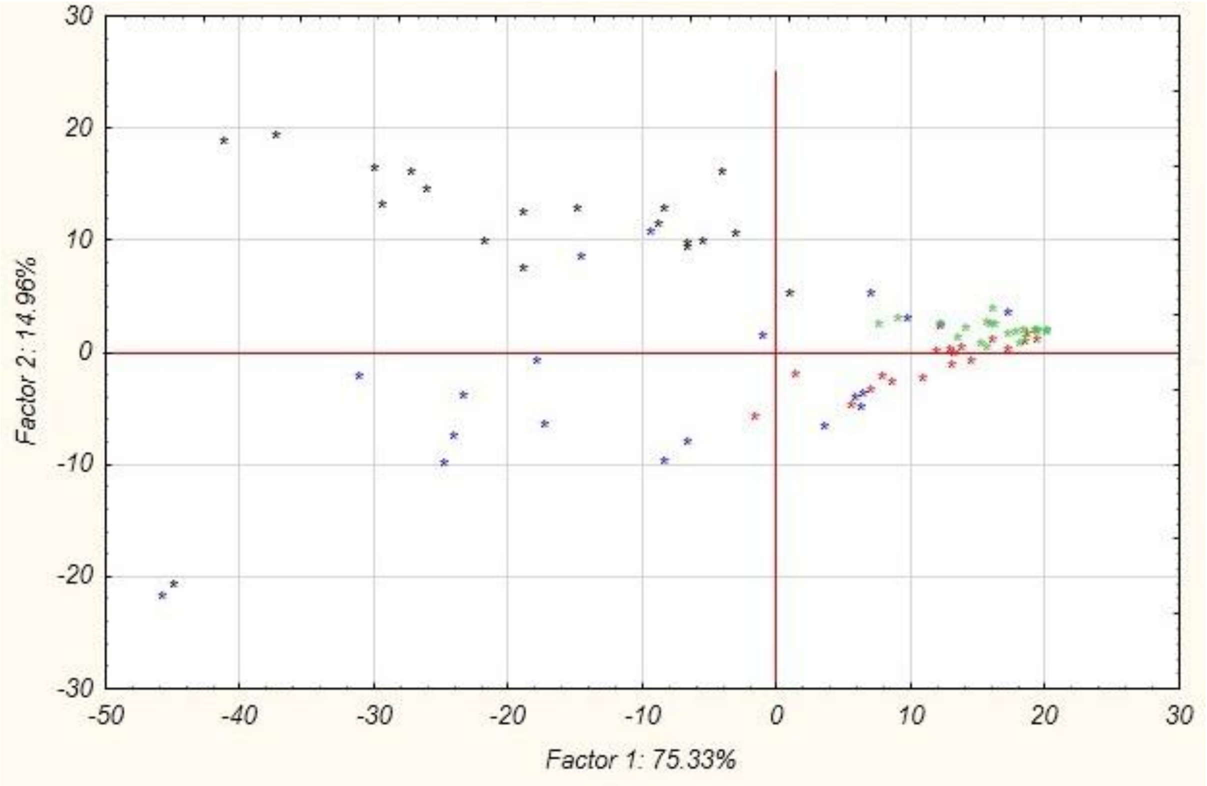

Figure S2. PCA analysis of 14 nm and 20 nm AuNP spectra.

PCA score plot of Factor 1 vs Factor 2 for the spectra obtained for 14 nm singularly dispersed AuNPs (red), 14 nm aggregated AuNPs (blue), 20 nm singularly dispersed AuNPs (green), and 20 nm aggregated AuNPs (black).

3. Supplementary Information on Spectral Profiles

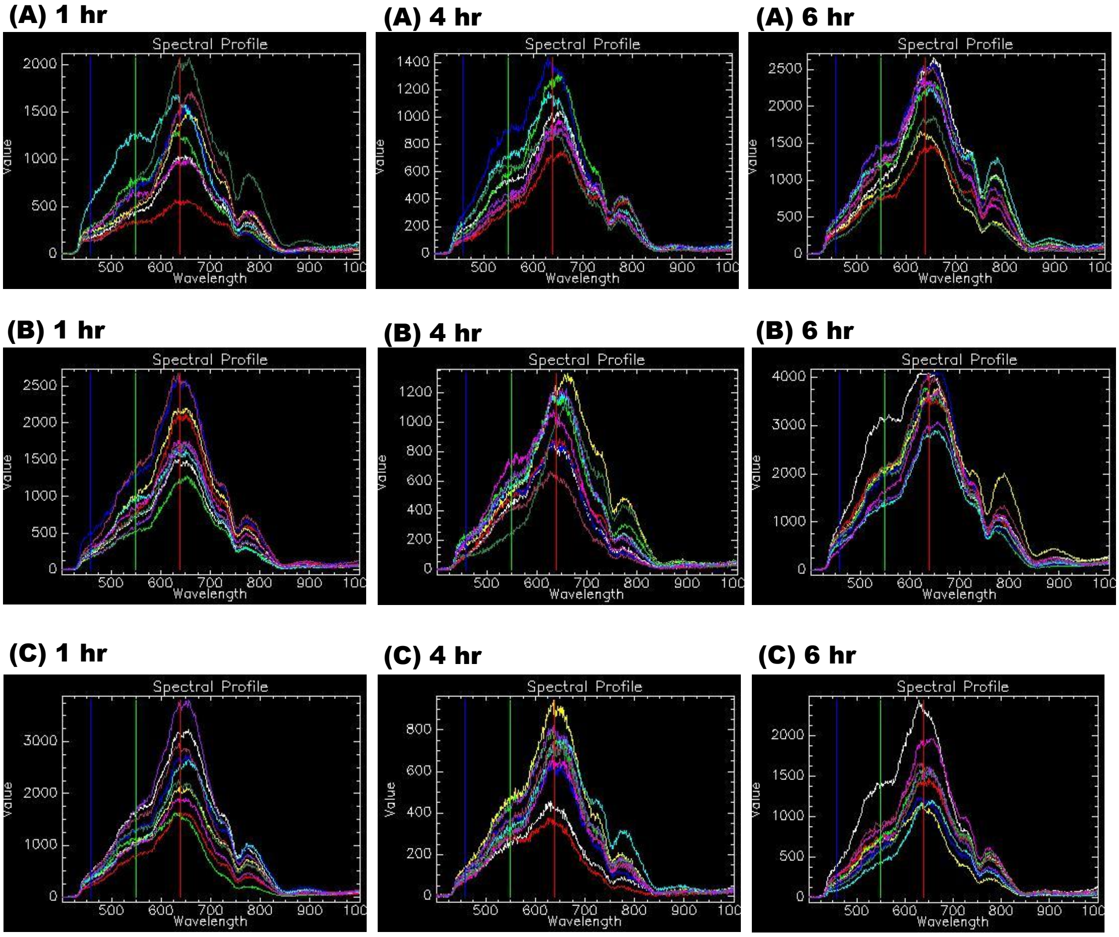

Figure S3. Spectral profiles of 14 nm AuNPs following cellular uptake.

Spectral profiles of 14 nm AuNPs were collected following uptake in (A) BEAS-2B, (B) HEK 293, and (C) CHO cells after 1 hr, 4hrs, or 6 hrs. Each image represents the spectral profiles of 10 randomly selected nanoparticles, and each coloured line represents the spectrum from a single pixel.

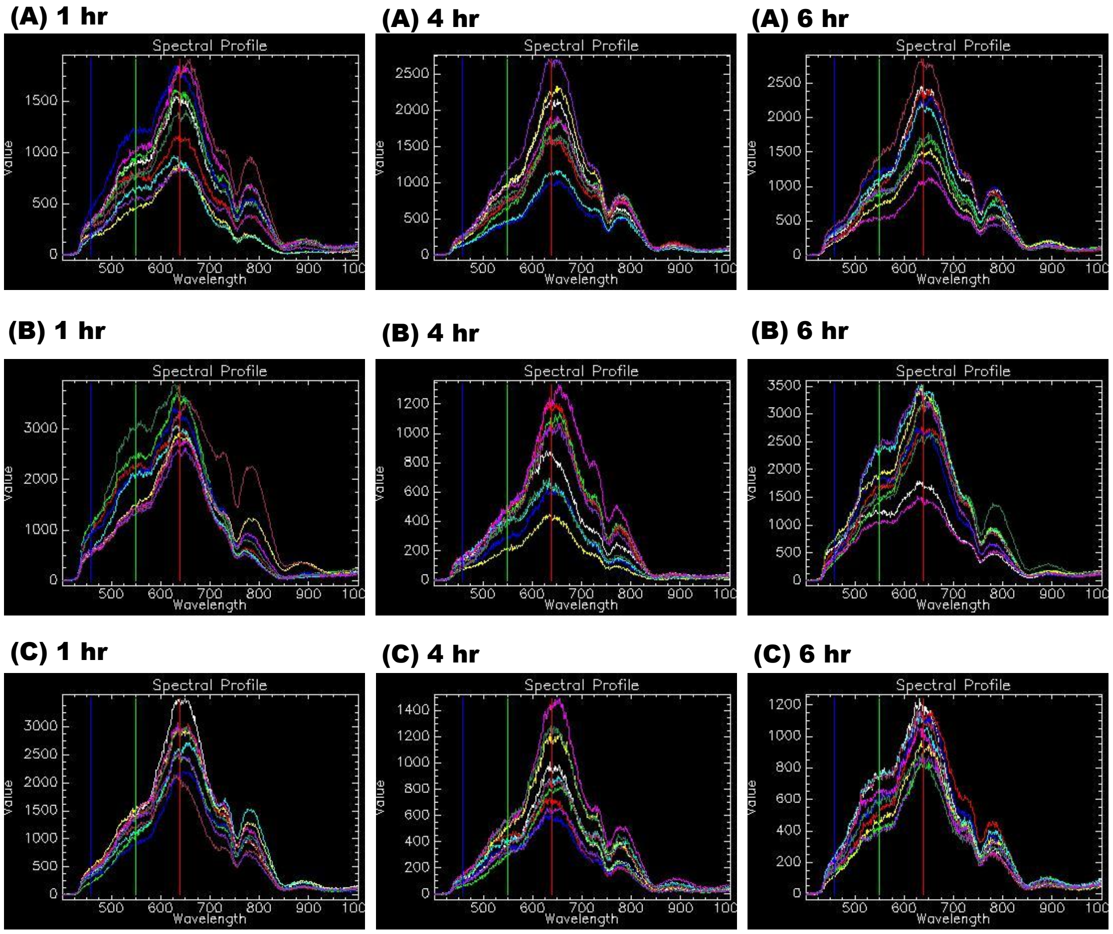

Figure S4. Spectral profiles of 20 nm AuNPs following cellular uptake.

Spectral profiles of 20 nm AuNPs were collected following uptake in (A) BEAS-2B, (B) HEK 293, and (C) CHO cells after 1 hr, 4hrs, or 6 hrs. Each image represents the spectral profiles of 10 randomly selected nanoparticles, and each coloured line represents the spectrum from a single pixel

2. DLS on NPs in culture media

The composition of cell culture media differs and therefore the hydrodynamic size of the nanoparticles in each was ascertained.

Methods

Dynamic Light Scattering (DLS) was performed using Malvern Instruments' Zetasizer Nano ZS. Nanoparticles were resuspended at 1 nM in either RPMI or Ham's F12 culture medium. DLS measurements were taken immediately, and after 4 hr and 6 hr incubations. T-tests were conducted using Statistica version 12 to determine if any significant increase in average size occurred.

Hydrodynamic size of nanoparticles in culture media

Average sizes of the AuNPs in culture medium are shown in Table S1. Size was determined based on scattered intensity. The average size of the 14 nm AuNP in culture media ranges from 57 nm to 67 nm, whilst that of the 20 nm AuNP in culture media ranges from approximately 69 nm to 71 nm. The only significant time-dependent increase ($p < 0.01$) of average size was observed with the 14 nm AuNP in Ham's F12 culture medium; in addition the size of the 14 nm AuNPs at 4 and 6 hrs was significantly different ($p < 0.01$) between culture media types. All samples showed high degrees of polydispersity. Since TEM data (Figure 2, main manuscript) showed that neither AuNP aggregated when suspended in culture medium, it can be deduced that the observed measurements are due to the formation of a protein corona.

Comparable bimodal distributions were observed for all samples, a representative figure of which is shown in Figure S5. The major mode accounts for approximately 80% of the intensity observed in all samples. The smaller peak observed lies between approximately 7 nm and 11 nm. This smaller peak has been observed in previous studies where DLS was used to measure the size of gold nanoparticles [3-4]; in both studies the authors regard this peak as a false peak, possibly due to rotational motion of nonspherical particles [4].

Table S1. Average hydrodynamic size of 14nm and 20nm AuNPs in culture medium as determined by DLS.

	Average size in nm by Intensity (Std dev)		
	0 hour	4 hour	6 hour
14 nm AuNP (RPMI)	58.3 (0.85)	59.9 (0.60)	58.8 (2.44)
14 nm AuNP (Ham's F12)	57.0 (0.90)	67.0 (0.99)	66.8 (0.96)
20 nm AuNP (RPMI)	68.7 (8.02)	69.0 (1.43)	71.2 (0.83)
20 nm AuNP (Ham's F12)	69.4 (6.27)	70.4 (5.15)	67.2 (1.03)

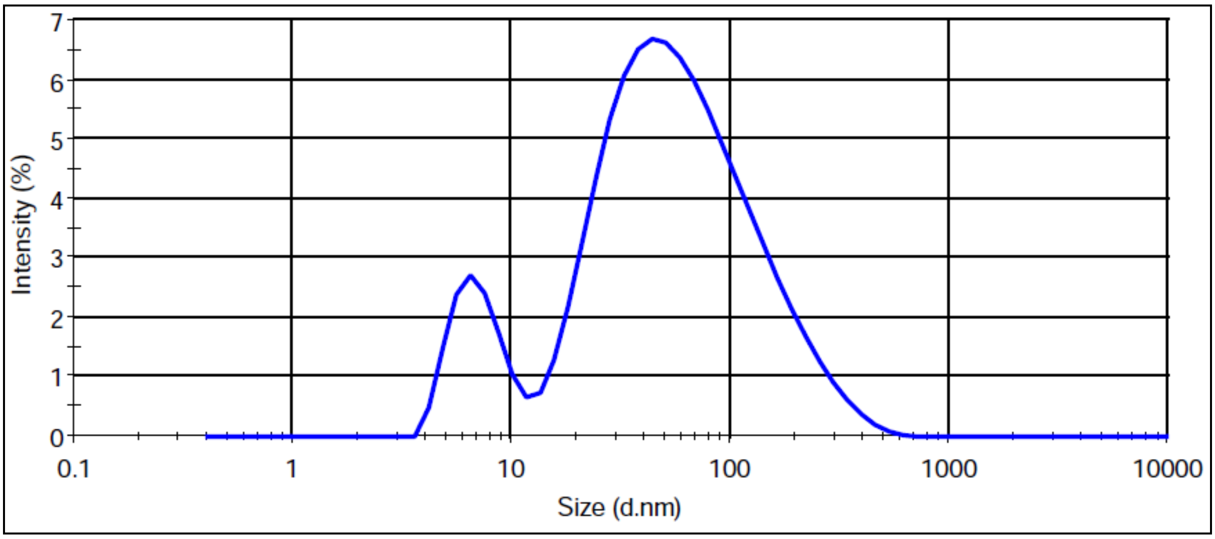

Figure S5. **Representative image of the bimodal distribution obtained from DLS.**

The size distribution, by intensity, of 20 nm AuNPs in RPMI culture medium is shown as a demonstration of the bimodal distribution obtained for all samples.

References

1. Chang C-I, Zhao X-L, Althouse MLG, Pan JJ: **Least Squares Subspace Projection Approach to Mixed Pixel Classification for Hyperspectral Images.** *IEEE Transactions on Geoscience and Remote Sensing* 1998, **36:**898-912.
2. Suganthy M, Ramamoorthy P: **Principal Component Analysis Based Feature Extraction, Morphological Edge Detection and Localization for Fast Iris Recognition.** *Journal of Computer Science* 2012, **8:**1428-1433.
3. Montes-Burgos I, Walczyk D, Hole P, Smith J, Lynch I, Dawson K: **Characterisation of nanoparticle size and state prior to nanotoxicological studies.** *Journal of Nanoparticle Research* 2010, **12:**47-53.
4. Khlebtsov BN, Khlebtsov NG: **On the Measurement of Gold Nanoparticle Sizes by the Dynamic Light Scattering Method.** *Colloid Journal* 2011, **73:**118-127.
